# Cell-Mediated Immunity to AAV Vectors, Evolving Concepts and Potential Solutions

**DOI:** 10.3389/fimmu.2014.00350

**Published:** 2014-07-23

**Authors:** Etiena Basner-Tschakarjan, Federico Mingozzi

**Affiliations:** ^1^Department of Hematology, The Children’s Hospital of Philadelphia, Philadelphia, PA, USA; ^2^University Pierre and Marie Curie, Paris, France; ^3^Genethon, Evry, France

**Keywords:** AAV vectors, T cell responses, gene therapy, immunogenicity, immune modulation

## Abstract

Adeno-associated virus (AAV) vectors are one of the most efficient *in vivo* gene delivery platforms. Over the past decade, clinical trials of AAV vector-mediated gene transfer led to some of the most exciting results in the field of gene therapy and, recently, to the market approval of an AAV-based drug in Europe. With clinical development, however, it became obvious that the host immune system represents an important obstacle to successful gene transfer with AAV vectors. In this review article, we will discuss the issue of cytotoxic T cell responses directed against the AAV capsid encountered on human studies. While over the past several years the field has acquired a tremendous amount of information on the interactions of AAV vectors with the immune system, a lot of questions are still unanswered. Novel concepts are emerging, such as the relationship between the total capsid dose and the T cell-mediated clearance of transduced cells, the potential role of innate immunity in vector immunogenicity highlighted in preclinical studies, and the cross talk between regulatory and effector T cells in the determination of the outcome of gene transfer. There is still a lot to learn about immune responses in AAV gene transfer, for example, it is not well understood what are the determinants of the kinetics of activation of T cells in response to vector administration, why not all subjects develop detrimental T cell responses following gene transfer, and whether the intervention strategies currently in use to block T cell-mediated clearance of transduced cells will be safe and effective for all gene therapy indications. Results from novel preclinical models and clinical studies will help to address these points and to reach the important goal of developing safe and effective gene therapy protocols to treat human diseases.

## Introduction

Several clinical studies have shown long-term correction of the disease phenotype following gene transfer (1–9). To attain this goal, two main approaches have been used, one using an integrating viral vector (typically retroviral or lentiviral) to introduce the therapeutic gene *ex vivo* into an autologous stem cell (10), the other transferring the gene into a post-mitotic cell *in vivo* (11).

Viral vectors derived from adeno-associated virus (AAV) have become the tool of choice for *in vivo* gene transfer, mainly because of their superior efficiency *in vivo* (11), their tropism for a broad variety of tissues, and their excellent safety profile. Therapeutic efficacy following AAV vector gene transfer was documented in several preclinical studies and, over the past decade, some of these results were successfully translated to the clinic, leading to some of the most exciting results in the field of gene therapy (11). The recent market approval of the first AAV-based gene therapy product in Europe (12, 13) constitutes additional evidence that the field is progressing from proof-of-concept studies toward clinical development.

However, human studies also highlighted some of the limitations of *in vivo* gene transfer with AAV vectors, which were not entirely identified in preclinical studies. In particular, it has been shown that immune responses triggered by AAV vector-mediated gene transfer may constitute an important obstacle to long-term therapeutic efficacy and a safety concern.

Over the past 10 years, gene therapists have struggled with the issue of immunogenicity of AAV vectors. The initial lack of animal models recapitulating the findings in human trials (14–16) has made clinical observation crucial to understand the interactions between AAV vectors and the immune system. While recent work shows that it is possible to model human T cell responses to the AAV capsid in mice (17), it is likely that human studies will remain the main source of knowledge on immune responses in gene transfer. In this review article, we will summarize the progress that has been made in the understanding of T cell responses in AAV vector-mediated gene transfer focusing on human studies, we will discuss the current state of knowledge, and we will describe some of the proposed strategies to modulate AAV vector immunogenicity.

## Wild-Type AAV and Recombinant AAV Vectors

Adeno-associated virus are small non-enveloped viruses with a single-stranded DNA genome of ~4.7 kb composed by the *rep* and the *cap* genes, which encode for the proteins involved in the life cycle of the virus. The *rep* gene is involved in the virus replication, while the *cap* gene encodes for the three structural proteins, VP1, VP2, and VP3, which form the viral capsid with a stoichiometry of 1:1:10, respectively (18), and for the assembly activating protein, which targets newly synthesized capsid proteins to the nucleolus and participates to their assembly into an icosahedral capsid (19). The *rep* and *cap* genes are flanked by two inverted terminal repeats (ITRs) that are needed as signals for packaging of the genome into the viral capsid (20). AAV is not autonomously replicating as its replication cycle depends on the coinfection with a helper virus such as adenovirus or herpes simplex; this explains why AAV was first isolated as a contaminant of an adenovirus preparation (21). The exposure to AAV and helper virus may account for the generation of both antibody and memory T cell response to AAV. AAV has never been associated with any known illnesses in humans with the exception of reports of association between spontaneous abortions and AAV infection (22). AAV vectors are derived from wild-type AAV by replacing all the viral coding sequences in the genome with an expression cassette for the transgene of interest. The ITRs are the only viral sequences retained in AAV vectors.

## AAV Vector:Host Interactions

**Figure 1:**
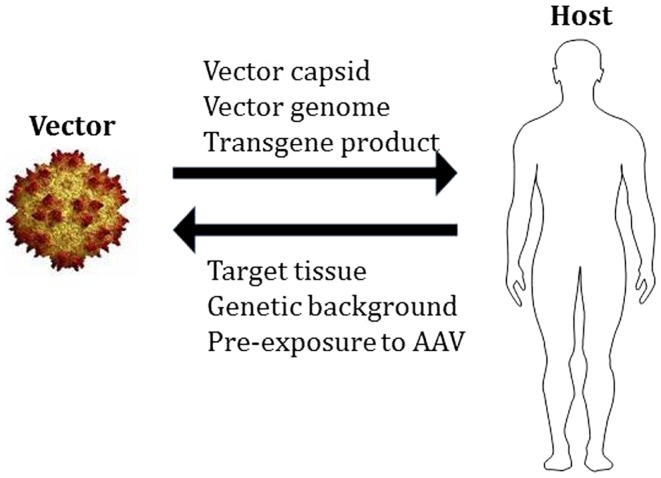
**Adeno-associated virus vector:host interactions**. The components of AAV vectors interact with the host in determining the outcome of gene transfer. For the AAV vector, the total capsid dose, the genome (single-stranded vs. self-complementary, CpG content, etc.), and the transgene product (to which the host may not be tolerant), represent some of the elements that could contribute to vector immunogenicity. On the host side, the characteristics of the target tissue (immunoprivilege, presence of inflammation, tolerogenic properties, etc.), the genetic background of the host (underlying disease-causing mutation, HLA haplotype, etc.), and the pre-exposure to the wild-type virus, can contribute to shape the overall response to vector delivery.

Like other viral gene transfer vectors, AAV vectors are complex biological therapeutics, and the outcome of gene transfer is dependent on the interactions between vector- and host-related components (Figure 1). These interactions occur at multiple levels, starting from the innate recognition of the vector capsid and DNA genome (23), to the development of humoral (24) and cell-mediated adaptive responses to the capsid and/or the transgene product.

### AAV vector capsid

At the immunological levels, the AAV vector capsid is a replica of the wild-type virion, therefore, the vector capsid antigen is expected to be recognized by T cells like the wild-type AAV capsid antigen. Despite this important similarity, however, vector administration differs from natural infection as it involves the introduction of large numbers of viral particles into an organism, several logs more than in a natural infection (25), via routes substantially different from the natural route of exposure to AAV (i.e., mainly via the airways for wild-type AAV), and does not involve active replication of the virus or presence of a helper virus, as AAV vectors are delivered as preformed particles. These differences are also characteristic of other viral gene transfer platforms, however, unique features of AAV (e.g., their lower immunogenicity compared to adenovirus) may account for the outcome of AAV gene transfer in humans.

Because AAV vectors are non-replicating, it is generally assumed that the AAV capsid remains immunologically detectable within a transduced cell only for a defined period of time, which may vary from few weeks to several months, depending on the target tissue. Experiments in primates in the context of liver gene transfer suggest that the capsid is still detectable by the immune system several weeks following administration (26), indicated by the fact that interruption of immunosuppression (IS) 8 weeks after gene transfer results in a spike in anti-AAV antibodies. In dogs, intact capsid was detectable by electron microscopy in the retina up to 6 years following gene transfer (27), and a similar result was recently obtained in human muscle, in which immunostaining for capsid particles showed a positive signal 12 months following vector administration (28). Finally, results in humans undergoing AAV8 gene transfer for hemophilia B suggest that the capsid antigen in transduced hepatocytes is still recognizable by capsid-specific T cells 8–9 weeks following gene transfer (9).

Why the capsid persists for such a long period of time is not clear at this point. There are several factors that may influence the persistence of viral particles in a tissue, among them the vector serotype and dose administered, the degree of vascularization of the tissue target of transduction, the number of particles introduced in a single injection site, and the possible immune response to the vector itself, which may increase the rate of clearance of the antigen. *In vitro* studies attempting to determine the half-life of the capsid in transduced cells have been largely unsuccessful in predicting the fate of the capsid, mainly because of the poor infectivity of most serotypes in cell lines (29).

### Vector DNA genome

Poorly defined until recently, the *in vitro* and *in vivo* interactions of both the DNA genome and the capsid of AAV vectors with the innate immune system were recently described by several groups (30–35). Additionally, recently published results indicate that the removal of CpG from the vector genome may contribute to reduce the potential immunogenicity of the transgene product (36). While these studies suggest that innate immunity to AAV vectors may trigger adaptive immune responses to the capsid or to the transgene product, there is no evidence that AAV vector administration in humans result in acute inflammatory reactions, although detailed studies in subjects undergoing AAV gene transfer remain to be performed.

### Transgene product

Unlike the AAV vector capsid, which is not synthesized by infected cells, the transgene product is expressed for a long time after target tissue transduction (37–39). Vector-encoded transgene product may be recognized as a foreign antigen, especially if the recipient of gene transfer is not tolerant to the protein encoded by the vector, thus triggering immune responses that can result in production of transgene-specific neutralizing antibodies (40) or triggering of T cell responses directed against transgene-expressing transduced cells (41).

Preclinical studies suggest that the tissue target of transduction plays a fundamental role as determinant of transgene immune responses in gene transfer. For example, preclinical studies of intramuscular delivery of AAV vectors suggest that this approach carries a higher risk of triggering immune responses to the transgene (42) compared to other tissues such as the liver (43), and that the underlying disease-causing mutation is a major determinant of the risk of developing an immune response to the therapeutic transgene product following AAV-mediated gene transfer to the muscle (44). Conversely, delivery of AAV vectors to the muscle via the vasculature seem to reduce considerably the immunogenicity of the therapeutic transgene (45–47), suggesting that the more widespread and uniform transduction of muscle achieved with this route of vector delivery lowers transgene immunogenicity. Clinical results accumulated thus far on intramuscular delivery of AAV vectors seem to indicate that the approach is safe, as no subject enrolled in trials of intramuscular gene transfer developed anti-transgene immunity (13, 48, 49). Transgene-specific cell-mediated immunity was documented in only one study in which pre-existing immunity to dystrophin seemed to trigger the expansion of dystrophin-specific CD8^+^ T cell clones (41).

Differently form muscle, a number of studies showed that expression of a transgene in the liver is associated with induction of antigen-specific tolerance (43, 50–59). This was demonstrated for several antigens and in various animal models, including animals that were first immunized and then tolerized against the same antigen used for immunization using liver gene transfer (52, 55, 56).

Results from clinical gene transfer studies seem to support the hypothesis that AAV vector-mediated liver gene transfer is associated with tolerance, as no subject dosed with AAV vectors in the liver developed an immune response directed against the transgene product, including individuals carrying *null* mutations in the disease-causing gene (9, 60).

Lastly, one important point to keep in mind when discussing transgene immunity is that most of the clinical experience to date derives from studies in which cross-reactive immunologic material (CRIM) positive subjects and subjects with prior exposure to the therapeutic protein [e.g., recombinant or plasma-derived factor IX (FIX) for hemophilia B patients] were enrolled (9, 48, 60, 61). These categories of subjects are at lower risk of developing immune responses to the donated therapeutic gene, thus a careful assessment of the immunogenicity of the transgene in gene transfer will be necessary before enrolling previously untreated patients in gene transfer trials. Finally, also the disease state of the organ targeted with gene transfer can influence the magnitude of the immune responses observed following AAV vector-mediated gene transfer (62–64).

## Capsid Antigen Intracellular Processing and MHC Class I Presentation

Cell transduction with AAV vectors begins with binding of the virion to the cell surface proteins and carbohydrates, an event that is followed by endocytosis. The receptor and co-receptors used for cell entry vary among serotypes, although the receptors for all serotypes have not been identified yet. The steps of endosomal escape, nuclear transport, and vector uncoating are not completely understood in terms of timing or mechanism, this in part due to their complexity and the large number of pathways and compartments potentially involved in the process of cell transduction. The intracellular fate of AAV2 has been the most extensively investigated, because several cell lines are readily transduced by this serotype and because monoclonal antibodies recognizing AAV2 intact particles and capsid proteins were the first available. Of interest to this review article is the ability of the capsid antigen to be processed and cross-presented on MHC class I (MHC I) by transduced cells (e.g., hepatocytes). Since the initial findings on the immunogenicity of AAV2 vectors in human trials (60), the ability of replication-deficient AAV vectors to gain access to MHC I via cross-presentation has been object of debate, and several alternative hypothesis were formulated to explain why AAV2 vectors were immunogenic in humans but not in animal models. These included the possibility of preferential uptake of certain AAV serotypes by dendritic cells, the expression of *Cap* sequences packaged into AAV vectors, and the expression of alternative open reading frames within the transgene cDNA, which would generate aberrant proteins recognized as offending antigens by the host immune system [reviewed in Ref. (65)]. Several years of studies in preclinical models and, most importantly, in human trials (9) helped developing a better understanding of immune responses to AAV vectors, supporting the idea that capsid-specific T cell responses were responsible for clearance of transduced cells.

At the intracellular level, it has been shown that the capsid is substrate for ubiquitination (66, 67), and that proteasome inhibitors, or mutation of surface exposed tyrosine residues that normally undergo phosphorylation and ubiquitination, enhance transduction by enhancing nuclear uptake of virus (68, 69). Data supporting the hypothesis that the AAV capsid antigen is processed by the proteasome and presented on MHC I come from CTL assays performed using AAV-transduced human hepatocytes as targets, and HLA-matched capsid-specific CD8^+^ T cells as effectors (70). The fact that target cell lysis can be inhibited specifically using a capsid antigen-specific soluble T cell receptor (TCR) to block T cell access to the target cell (70) further confirms that the AAV capsid antigen is processed by transduced cells and presented on MHC I. Presentation of antigen in the context of MHC I was also demonstrated using a T cell line engineered to express luciferase when recognizing the capsid antigen (71). In this study, levels of antigen presentation were directly correlated with the multiplicity of infection used in the assay, and the proteasome inhibitor bortezomib blocked antigen presentation. These results are in agreement with results from clinical studies, which suggest the existence of a correlation between vector dose and magnitude of T cell responses to the capsid.

Recent work from Li et al. (72) indicates that the endosomal escape of vector is not only the pivotal step in cell transduction but is also fundamental for capsid antigen presentation. While this is not completely surprising, the study also indicates that empty capsids, AAV particles that fail to incapsidate a DNA genome, are less likely to be presented onto MHC I. This result is somewhat in contrast with previous data showing that empty capsids do flag transduced cells for T cell recognition (70). One possible explanation for this difference is that the two studies used different strategies to detect antigen presentation: ovalbumin TCR transgenic T cells and a capsid carrying the ovalbumin SIINFEKL epitope in one study (72), or human peripheral blood mononuclear cells (PBMCs) expanded against a the native AAV epitope VPQYGYLTL to track presentation of native AAV2 capsid antigen (70). Immunogenicity of empty vs. full AAV capsids is an important pending question for the field, particularly given the fact that empty capsids may play an important beneficial role in allowing for vector transduction in the presence of neutralizing antibodies (73).

Although there is no direct evidence that intracellular processing and presentation of AAV onto MHC I differ among serotypes, anecdotal evidence supporting this hypothesis comes, for example, from the observation that the administration of AAV2 and AAV8 vectors to the liver of humans at similar doses results in different kinetics of activation cytotoxic T cell immunity against the transduced hepatocytes (9, 60, 74). Whether these differences can be ascribed to the vector serotype only is unknown, as *in vitro* studies comparing AAV serotypes have been challenging due to differences in efficiency of AAV transduction among serotypes. Conversely, *in vivo* preclinical studies show somewhat contrasting results, some suggesting for example that AAV2 and AAV8 have identical kinetics of T cell induction (75), and others showing that AAV2 and AAV8 do differ in their ability of triggering T cell proliferation (76). Serotype-specific differences are also supported by data generated with an adoptive T cell transfer model in mice, indicating that AAV8 remains immunologically detectable longer than AAV2 following systemic gene transfer to target the liver (17, 76).

In conclusion, several studies identified the key intracellular AAV trafficking steps that are involved in cell transduction and capsid antigen presentation. Whether there is one leading pathway for antigen cross-presentation or rather multiple alternative pathways concur to MHC I presentation (77) of capsid remains to be defined. Most importantly, the significance of *in vitro* findings has to be proven *in vivo*, as kinetics and pathways might differ substantially in living organisms compared to cell lines.

## T Cell Responses to AAV in Human Studies

Several studies on the seroprevalence of AAV in humans suggest that exposure to the wild-type virus mostly occurs early in life (78–80). Similarly, monitoring of T cell reactivity to the AAV2 capsid conducted in humans undergoing splenectomy for non-malignant indications shows that about two-thirds of adults >25 year-old carry a pool of T cells that can produce IFN-γ in response to AAV2 capsid peptides, while only a small proportion of subjects <5 years old present T cell reactivity to the capsid (81). Veron et al. (82) conducted a similar survey for AAV1 in PBMCs from healthy donors. In this study, an overall frequency of respondents of about 30% was documented. The difference in frequency of subjects carrying capsid-specific T cells in the two studies may be due to the fact that reactive T cells fail to circulate in peripheral blood at high frequency [as previously suggested in Ref. (74)], or to the different restimulation protocols used (a peptide library was used in the AAV2 study vs. lentiviral vectors expressing capsid were used in the AAV1 study), or else it may reflect inherent differences in the frequency of subjects exposed to AAV2 vs. AAV1.

Two aspects of capsid T cell reactivity are worth noting, the first is that the high degree of conservation of the AAV capsid amino acid sequence (83) results in a high degree of cross-reactivity of T cell responses across serotypes (74). The second aspect is that B and T cell responses to AAV seem to be uncoupled, as subjects positive for anti-AAV antibodies may not present detectable T cell reactivity to the capsid and, vice versa, subjects with detectable T cell reactivity to AAV in PBMC have lower anti-capsid antibody titers (82). This suggests that, following exposure to AAV, certain individuals may develop a Th1 response to the antigen, while others develop a predominantly Th2 response. Future studies will be required to clarify the relationship between B and T cell responses to AAV in the context of the natural infection with the virus.

### Liver-directed gene transfer

The importance of T cell immunity to the AAV capsid in terms of both safety and efficacy of AAV gene transfer in humans was initially evidenced in the first clinical trial in which an AAV2 vector was introduced into the liver of severe hemophilia B subjects (60). In this study, upon AAV gene transfer to liver, two subjects developed transient elevation of liver enzymes and loss of FIX transgene expression around week 4 post vector delivery due to the immune rejection of transduced hepatocytes mediated by capsid-specific CD8^+^ T cells (74).

While conceptually these findings are not surprising, as antiviral immunity is expected to recognize virus infected cells and clear them, this was the first instance in which a cytotoxic immune response directed against the AAV capsid was observed in the context of gene transfer. Preclinical animal studies failed to predict or to recapitulate the findings in humans, and initial attempts to model the induction of destructive T cell responses in mice have been mostly unsuccessful until recently (17).

The recent findings in a clinical trial of AAV8 gene transfer of FIX to the liver of subjects affected by severe hemophilia B (9) confirmed results in the previous AAV2 trial and supported the hypothesis that AAV capsid antigen is processed and presented onto MHC I by vector-transduced hepatocytes where it is recognized by capsid-specific CD8^+^ T cells, leading to clearance of vector-transduced hepatocytes, transaminase elevation, and loss of FIX transgene expression.

In particular, in the AAV8-FIX study vector administration resulted in the activation of capsid-specific CD8^+^ T cells, with an increase in liver enzymes that required intervention with corticosteroids detected in four out of six subjects (9, 84) from the high-dose cohort, who received 2 × 10^12^ vg/kg of vector, ~8–9 weeks after vector delivery. Results from this clinical study suggest that a short course of steroids, administered at the time of liver enzymes elevation and loss of transgene expression, can at least partially rescue transgene expression. This study also highlights differences in kinetics of T cell responses between the AAV2 and the AAV8 liver trials, as liver enzyme elevation in the AAV2 trial was observed around week 4 following vector delivery (60), as opposed to the 8- to 9-weeks in the AAV8 trial (9). It is not clear at this point what is the likely explanation for this difference.

One important emerging aspect of AAV capsid-driven capsid T cell reactivity in humans is that capsid-specific T cell responses seem to be detected in a dose-dependent fashion, a result consistent with published *in vitro* antigen presentation data (70). Above a certain threshold of capsid antigen dose, the activation of capsid-specific T cells results in loss of transduced hepatocytes. Whether at vector doses higher than those tested thus far all subjects will mount a T cell response that will result in loss of transgene expression, and whether steroids will effectively control capsid T cells at all vector doses, is not clear at this point. Several factors are like to influence the outcome of vector administration in humans in terms of T cell reactivity, thus complicating the interpretation of results, among them the HLA type of the subjects infused, the concomitant presence of inflammation in the target tissue, and other vector-specific features that may enhance immune responses to the vector, the transgene, or both.

### Gene transfer to the muscle

Adeno-associated virus vector-induced T cell immunity is not unique to liver-directed gene transfer. Monitoring of capsid T cell responses has been performed in the context of several muscle-directed gene transfer clinical studies (41, 85–92) as well. In agreement with the findings in AAV liver gene transfer studies, results accumulated for muscle gene transfer suggest that the magnitude of T cell responses directed against the AAV capsid correlates with the dose of vector administered (65, 92). Following intramuscular AAV vector delivery, an increase in frequency of circulating reactive T cells in PBMC is observed at higher vector doses (91). In some cases, detection of capsid T cell activation in PBMC correlated with lack of transgene expression in vector injected muscle (89, 91); while in other studies the detection of capsid T cells in PBMC seemed to have no effect on transgene persistence (28).

Immunosuppression has been used in some of the muscle gene transfer studies conducted thus far to modulate capsid immunogenicity (13). Whether this helped the persistence of transgene expression is not completely clear due to the lack of readily detectable efficacy endpoints and the fact that IS itself complicates immunomonitoring as it is likely to modify capsid-directed T cell responses.

In a recent study of intramuscular gene transfer for α_1_-antitrypsin deficiency (92), vector administration was associated with detection of capsid-specific T cell reactivity and increase in serum creatine kinase in some subjects around day 30 post vector administration. Furthermore, activation of both CD4^+^ and CD8^+^ T cells in peripheral blood and T cell infiltrates in muscle biopsies were detected. T cell reactivity against the AAV capsid antigen did not seem to result in clearance of transduced muscle fibers or loss of transgene expression, a finding that may be explained by the fact that significant amounts of CD4^+^CD25^+^FoxP3^+^ regulatory T cells (Tregs) were found in muscle biopsies (28). Whether the local induction of Tregs is a phenomenon unique to muscle or to the α_1_-antitrypsin transgene (93) remains to be established. However, these findings indicate that proinflammatory and tolerogenic signals may be concomitantly elicited by vector administration and may concur in determining the outcome of gene transfer. Similarly, apoptosis of transgene reactive T cells in muscle has been documented in mice (94) and in humans (90), indicating that additional factors are involved in the modulation immunogenicity of vector and transgene other than the target organ alone.

The inability of collecting tissue biopsies in the AAV trials for hemophilia conducted thus far prevented investigators from comparing results between liver and muscle gene transfer, thus leaving open important questions on the relevance of tissue-specific features that may influence vector immunogenicity.

### Gene transfer to immunoprivileged body sites

Adeno-associated virus vectors have been administered to humans in several eye- (95) and brain- (96–102) targeted gene transfer trials. So far, doses tested in these immunoprivileged body compartments were lower compared to the doses tested in the liver or muscle trials, and they fail to elicit significant antibody or cell-mediated immune responses to capsid or transgene product. While these results strongly support the safety of gene transfer to eye and brain, future studies will help to understand whether the immune privilege is maintained at all vector doses, especially given the fact that some AAV vector serotypes can escape the blood–brain barrier (103), thus resulting in systemic exposure to the vector.

## Overcoming T Cell Immunity to AAV

More than 10 years have passed since the initial AAV2 FIX trial. The field of *in vivo* gene transfer with AAV vectors has progressed enormously thank to a multitude of preclinical and clinical studies that clarified the nature of anti-capsid immune responses and tested the efficacy of strategies aimed at preventing or modulating these responses. In this section of our review article, we will discuss some of these strategies, with particular emphasis on those already tested in the clinic.

### Reduce the total capsid antigen dose

Results from the AAV8 hemophilia trial (9, 84) suggest that lower vector doses may not trigger destructive T cell responses to the capsid. In this study, subjects who received vectors doses up to 6 × 10^11^ vg/kg did not experience increase in liver enzymes or loss of transgene expression, at the same time levels of FIX transgene were just above the threshold for therapeutic efficacy. Different strategies are being tested to maintain the total capsid dose low while increasing therapeutic efficacy. One is to use hyperactive variants of the therapeutic protein (104–106), or stronger promoter elements (107), or else codon-optimized transgenes (108, 109). All these maneuvers are either being tested in the clinic or about to enter clinical trials and results will be available soon.

Engineering the AAV capsid to prevent its presentation onto MHC I (17, 69) or administration of proteasome inhibitors (71) to block processing of capsid antigen are also strategies that have been proposed to reduce capsid antigen load following vector administration. These strategies are also associated with higher transduction efficiency in animal models (71, 110), thus they could potentially allowing to decrease the therapeutic dose; however, the role of alternative antigen presentation pathways should be taken into account when evaluating this strategy. Furthermore, the use of proteasome inhibitors like bortezomib is not devoid of potentially serious side effects (111).

The use of AAV vector preparations devoid of empty capsids is also being tested as a strategy to reduce the overall antigen load associated with gene transfer. While clearly this results in lower total amounts of capsid being administered, in the negative side it may render vectors more susceptible for antibody-mediated neutralization (73). The jury is still out on the role of empty capsids in gene transfer, with some reports suggesting that these particles are not immunogenic (72), others arguing that they are detrimental for transduction efficiency (112), and recent reports showing that they are beneficial in the presence of anti-AAV neutralizing antibodies (73).

### Use transient immunosuppression

One potential advantage of the use of IS the context of AAV gene transfer is that, differently from organ transplant or autoimmune disease, the duration of the intervention is expected to be relatively short (9). Initial studies of gene transfer with IS favored the idea of treating subjects upfront, starting just before or at the time of vector administration (26, 58, 113–116). The advantage of this approach is that any immune response occurring after vector deliver would be prevented, the main disadvantage consist in the fact that IS would prevent efficient immunomonitoring, thus would not allow to study the nature and kinetics of immunity to AAV. Additionally, treating all subjects with IS may not be necessary, as some may not develop immunity to the vector (9, 84).

Aside from the obvious higher risk of infection, IS may also influence induction of Tregs (58), which are fundamental for the maintenance of transgene tolerance in liver (43) gene transfer and seem to play an important role in muscle gene transfer (28). Recent reports show impaired transduction levels under IS with mycophenolate mofetil (113), others report a longer plasma half-life of vector in non-human primates receiving IS (114). These are all evidences that interactions of IS with viral gene transfer are quite complex and may result in unexpected findings.

The use of transient IS with steroids at the time of liver enzyme elevation is a relatively novel concept in the field of gene transfer that has been successfully adopted in the AAV8 hemophilia trial. In this study, a mild elevation of liver enzymes has been immediately treated with high-dose prednisolone, resulting in the prevention of what appeared to be an immune-mediated clearance of AAV8 transduced hepatocytes. The main advantage of the approach is that not all subjects were exposed to IS, in fact only those who had increased liver enzymes received prednisolone. The challenge of the approach is that endpoints of liver (or other organ) immunotoxicity may not be always obvious, making a targeted intervention hard. In these latter cases, upfront IS may be necessary; however, the key question revolves around the timing and the duration of this approach. Additionally, steroids may not work in some subjects or at high vector doses. These concerns will be addressed in future studies.

In conclusion, one important consideration about IS regimens and timing of intervention is about the immune state of infused subjects. Some individuals may in fact be recently primed by natural infections with wild-type AAV, or else could have been previously enrolled in gene therapy protocols (in case of vector readministration). Immune responses to the vector capsid in these subjects may be expected to be faster and stronger, thus warranting particular attention.

### Can we tolerize subjects against the AAV capsid?

As an alternative to IS, the induction of Tregs specific to the AAV capsid can result in sustained expression of the transgene with no induction of destructive T cells.

MHC class II epitopes found in human IgG (117) have been described to induce proliferation of Tregs and suppress Th1 and Th2 immune responses (117–120). These peptides, named Tregitopes, have been used to modulate CD8^+^ T cell responses to AAV, resulting in suppression of cytotoxic responses against AAV-transduced cells and expansion of Tregs *in vitro*. Additional studies *in vivo* (118), in which Tregitopes were co-expressed with the capsid antigen, resulted in modulation of CD8^+^ T cell responses to the capsid antigen itself following adenoviral vector-mediated vaccination. While results *in vitro* suggest that the approach leads to antigen-specific tolerance (118), additional studies are needed to test if antigen-specificity is conserved *in vivo*, whether the approach is safe, and ultimately, how to translate this strategy to the clinic.

## Conclusion

Since the initial proof-of-concept studies, the field of *in vivo* gene transfer has progressed enormously. Experience from the clinical translation of AAV vector-based gene transfer strategies has highlighted the challenges and helped optimize the choice of the AAV vector serotype, the delivery methods, and has highlighted some of the limitations of the approaches tested. In particular, the interactions between the human immune system and all the components of gene therapy vectors seem to represent one of the major limitations to long-lasting therapeutic efficacy.

The small scale of gene transfer trials for rare diseases, the heterogeneity of vector serotypes, transgenes, and doses tested, and the ability of measuring the endpoints of therapeutic efficacy have been an obstacle to the advancement of knowledge. Furthermore, individual variability associated for example with HLA haplotype and with the underlying disease state also represents an added layer of complexity to data interpretation.

Gene therapists should not forget that immune responses triggered by gene transfer must be understood and studied as a complex network of interactions; as such, the outcome of gene transfer in immunological terms is influenced by both innate and adaptive immunity, which are influenced by the nature of the vector, transgene, route, etc. Future immunosurveillance studies conducted in clinical gene transfer studies will provide the basis for a better understanding of the determinants of T cell responses in AAV-mediated gene transfer, for example, the role of target tissue inflammation due to the underlying disease. Standardization of technologies used for monitoring of T cell responses (121, 122) will help to correlate them with clinical outcomes and, eventually, devise novel strategies around the issue.

Despite these challenges, the field has acquired critical information from the studies conducted thus far, which allowed to address the issue of vector immunogenicity. The results of these efforts are evident, long-term follow up data from AAV gene transfer trials show many examples of long-lasting therapeutic efficacy. And results accumulating suggest that once transgene expression is established, and immune responses avoided, multi-year therapeutic efficacy is a goal attainable in humans.

## Conflict of Interest Statement

Federico Mingozzi is inventor in patents describing the AAV vector technology and technologies to modulate immunity to AAV. Etiena Basner-Tschakarjan reports no conflicts of interest.
